# Elevated plasma levels of WAP four-disulfide core domain 2 as a potential prognostic biomarker for various cancers

**DOI:** 10.3389/fonc.2025.1614102

**Published:** 2025-07-14

**Authors:** Makoto Watanabe, Katsuaki Ieguchi, Nobuyuki Onishi, Takashi Shimizu, Ryotaro Ohkuma, Risako Suzuki, Emiko Mura, Nana Iriguchi, Tomoyuki Ishiguro, Yuya Hirasawa, Go Ikeda, Masahiro Shimokawa, Hirotsugu Ariizumi, Atsushi Horiike, Kiyoshi Yoshimura, Takuya Tsunoda, Mayumi Tsuji, Shinichi Kobayashi, Yuji Kiuchi, Satoshi Wada

**Affiliations:** ^1^ Department of Clinical Diagnostic Oncology, Clinical Research Institute for Clinical Pharmacology and Therapeutics, Showa Medical University, Tokyo, Japan; ^2^ Clinical Research Institute for Clinical Pharmacology and Therapeutics, Showa Medical University, Tokyo, Japan; ^3^ Department of Pharmacology, School of Medicine, Showa Medical University, Tokyo, Japan; ^4^ Pharmacological Research Center, Showa Medical University, Tokyo, Japan; ^5^ Division of Medical Oncology, Department of Medicine, School of Medicine, Showa Medical University, Tokyo, Japan

**Keywords:** WFDC2, biomarker, plasma, ELISA, cancer prognosis

## Abstract

**Background:**

Although WAP four-disulfide core domain 2 (WFDC2) is widely recognized as a diagnostic biomarker for ovarian cancer, its function in other cancer types remains unclear. The aim of this study was to evaluate the prognostic potential of WFDC2 across multiple cancers.

**Methods:**

Publicly available transcriptomic datasets were analyzed to compare WFDC2 mRNA expression in normal and tumor tissues across various cancer types. Kaplan-Meier survival analysis and Cox proportional hazards regression were used to assess the association between WFDC2 mRNA expression and overall survival (OS). Plasma WFDC2 levels were measured using enzyme-linked immunosorbent assay in healthy donors as well as patients with gastric, lung, colorectal, esophageal, and pancreatic cancers. Receiver operating characteristic (ROC) analysis was performed to evaluate the diagnostic performance of WFDC2. Kaplan–Meier analysis was conducted to evaluate the association between WFDC2 expression and OS. Cox proportional hazards regression analysis was performed to assess the prognostic significance of WFDC2 expression.

**Results:**

WFDC2 mRNA expression was significantly elevated in gastric cancer, lung adenocarcinoma (LUAD), esophageal carcinoma, and pancreatic ductal adenocarcinoma (p < 0.01); however, it was significantly lower in colorectal cancer (p < 0.005). Kaplan-Meier analysis indicated that elevated WFDC2 mRNA expression in LUAD was only significantly associated with prolonged OS (p = 0.017), whereas no significant associations were observed in other cancer types. Moreover, plasma WFDC2 levels were significantly higher in all cancer patient groups than in healthy donors (p < 0.0001). ROC analysis revealed potential diagnostic performance, with an area under the curve of 0.890 (95% CI: 0.844–0.936) for distinguishing patients with cancer from healthy donors. Subgroup analysis indicated diagnostic performance across all cancer types. Elevated plasma WFDC2 levels were significantly associated with shorter OS in esophageal cancer (p = 0.0226). Multivariate Cox regression analysis confirmed that plasma WFDC2 concentration was an independent prognostic factor in gastric (HR = 1.04, 95% CI: 1.00–1.07, p = 0.019) and esophageal cancers (HR = 1.08, 95% CI: 1.02–1.13, p = 0.006).

**Conclusion:**

Plasma WFDC2 levels demonstrated potential diagnostic performance across multiple cancers and were significantly associated with poor prognosis in gastric and esophageal cancers.

## 1 Introduction

Cancer remains one of the leading causes of death worldwide, accounting for millions of lives annually and affecting families and communities. The World Health Organization estimates that approximately 10 million people died from cancer in 2020 alone (1), underscoring the urgent need for effective detection and treatment strategies. The complexity of cancer biology, characterized by diverse genetic mutations and immune evasion (2), often results in diagnoses at advanced stages, leading to poor prognoses. Early detection is crucial for improving patient outcomes, as many cancers remain asymptomatic until reaching advanced stages. Although traditional imaging techniques and screening methods are valuable, their inherent limitations in detecting early-stage cancers underscore the necessity to identify reliable and specific biomarkers (3).

Among identified potential biomarkers, WAP four-disulfide core domain 2 (WFDC2), also known as human epididymis protein 4 (HE4), has emerged as a promising candidate for cancer detection and monitoring. Initially identified in the distal epithelium of the epididymis (4), where it likely functions as a protease inhibitor during sperm maturation (5, 6), WFDC2 has gained prominence as a diagnostic biomarker for ovarian cancer. Elevated serum WFDC2 levels significantly enhance diagnostic accuracy when combined with CA125, correlating strongly with disease presence and progression (6–8).

In addition to ovarian cancer, increased WFDC2 expression has been reported in lung cancer, suggesting its broader applicability in cancer diagnosis and management. Moreover, the correlation of WFDC2 expression with lung cancer progression underscores its potential for improving diagnostic strategies and informing treatment decisions (9–12). Similarly, in lung adenocarcinoma (LUAD), elevated WFDC2 expression has consistently been associated with better overall survival (OS), underscoring its potential as a prognostic biomarker (11). However, although WFDC2 is upregulated in LUAD, this same increase in expression is not observed in lung squamous cell carcinoma (LUSC), suggesting a tumor subtype-specific role (12). This differential expression highlights that WFDC2 has a complex role in lung cancer and its potential as a biomarker for specific cancer subtypes.

Recent studies have highlighted that WFDC2 is overexpressed in gastric cancer, where it is linked to poor prognosis and resistance to radiation therapy (13, 14). However, its potential as a biomarker in gastric cancer has not yet been extensively evaluated using enzyme-linked immunosorbent assay (ELISA)-based analysis of blood samples, leaving its clinical significance and biological functions largely unexplored. Similarly, WFDC2 has been implicated in drug resistance and poor prognosis in pancreatic cancer (13, 15), underscoring its role in cancer progression and treatment resistance. Furthermore, our previous research demonstrated that elevated WFDC2 expression in pancreatic cancer is associated with increased tumor aggressiveness and chemotherapy resistance (15), with overexpression correlating with significantly shorter OS.

In colorectal and esophageal cancer, serum WFDC2 levels have been proposed as a potential biomarker; however, their diagnostic capability remains limited and insufficient for clinical application (16, 17). Research into the biomarker potential and functional roles of WFDC2 in these cancers remains limited, with only a few studies available. Given these gaps, further investigation into WFDC2 as both a diagnostic and prognostic biomarker is essential for advancing cancer detection and treatment strategies. The aim of this study was to evaluate the potential of WFDC2 as a biomarker for cancer detection and its prognostic significance across various cancer types, using plasma samples to assess its applicability for early detection and therapeutic monitoring.

## 2 Materials and methods

### 2.1 Ethical considerations

The study protocol was approved by the Ethics Committee of Showa Medical University School of Medicine (Approval No. M2776), and all research procedures were conducted in accordance with the principles of the Declaration of Helsinki.

### 2.2 Sample collection

This study was conducted on a consecutive cohort of 158 patients diagnosed with gastric cancer, lung cancer, colorectal cancer, esophageal cancer, and pancreatic cancer at Showa Medical University Hospital between February 8, 2019, and February 6, 2023. Blood samples were collected prior to the initiation of any cancer-specific treatment from these patients, along with 20 healthy donors. The patient group included 39 individuals diagnosed with gastric cancer, 51 with lung cancer, 30 with colorectal cancer, 31 with esophageal cancer, and seven with pancreatic cancer. Among the 158 patient samples, 148 were classified as Stage IV, nine as Stage III, and one as Stage II. All participants, including the healthy donors and patients with cancer, provided informed consent prior to sample collection. Plasma was separated using the BD P100 Blood Collection System (366422, BD Biosciences, Franklin Lakes, NJ, USA) according to the manufacturer**’**s protocol. The collected plasma was aliquoted and stored at -80°C for further analysis.

### 2.3 ELISA for WFDC2 levels

Plasma WFDC2 levels were measured using the Human HE4/WFDC2 DuoSet ELISA Development System (DY6274, R&D Systems, Minneapolis, MN, USA), according to the manufacturer’s instructions. Briefly, anti-WFDC2 capture antibodies were coated onto a 96-well plate overnight. The following day, the plates were washed with wash buffer (0.05% Tween 20 in Phosphate-Buffered Saline (PBS)) and blocked with 1% BSA in PBS for 1 h. Plasma samples were centrifuged at 3000 rpm at 4 °C for 10 min, and the supernatants were diluted 1:20 in 1% BSA in PBS. Following additional washes, 100 µL of the diluted plasma samples was added to the wells and incubated for 2 h. Following additional washes, biotinylated detection antibodies were added, followed by streptavidin-horseradish peroxidase (HRP) and TMB substrate solution. The reaction was terminated using 2 N sulfuric acid, and optical density (OD) was measured at 450 nm, with correction at 570 nm, using a BioTek Synergy HTX Multimode Reader. Unless otherwise specified, all steps were performed at room temperature.

### 2.4 Statistical analysis

All statistical analyses were conducted using JMP Pro 14.0 (SAS Institute Inc.). The Wilcoxon test, a non-parametric method, was applied to compare two independent groups. For comparisons involving three or more groups, the non-parametric Steel test was performed, with healthy donors serving as the control group. Receiver operating characteristic (ROC) curves were generated based on plasma marker levels from healthy donors and patients with cancer. Survival was defined as the period from sample collection to cancer-specific death, with patients who remained alive or were transferred considered censored. Survival data were updated as of November 2024. Survival probabilities were assessed using the Kaplan-Meier method and evaluated using the log-rank test. Cox proportional hazards regression was used for univariate and multivariate analyses. All statistical tests were two-tailed, and statistical significance was set at p < 0.05.

## 3 Results

### 3.1 WFDC2 mRNA expression in normal and tumor tissues

**Figure 1:**
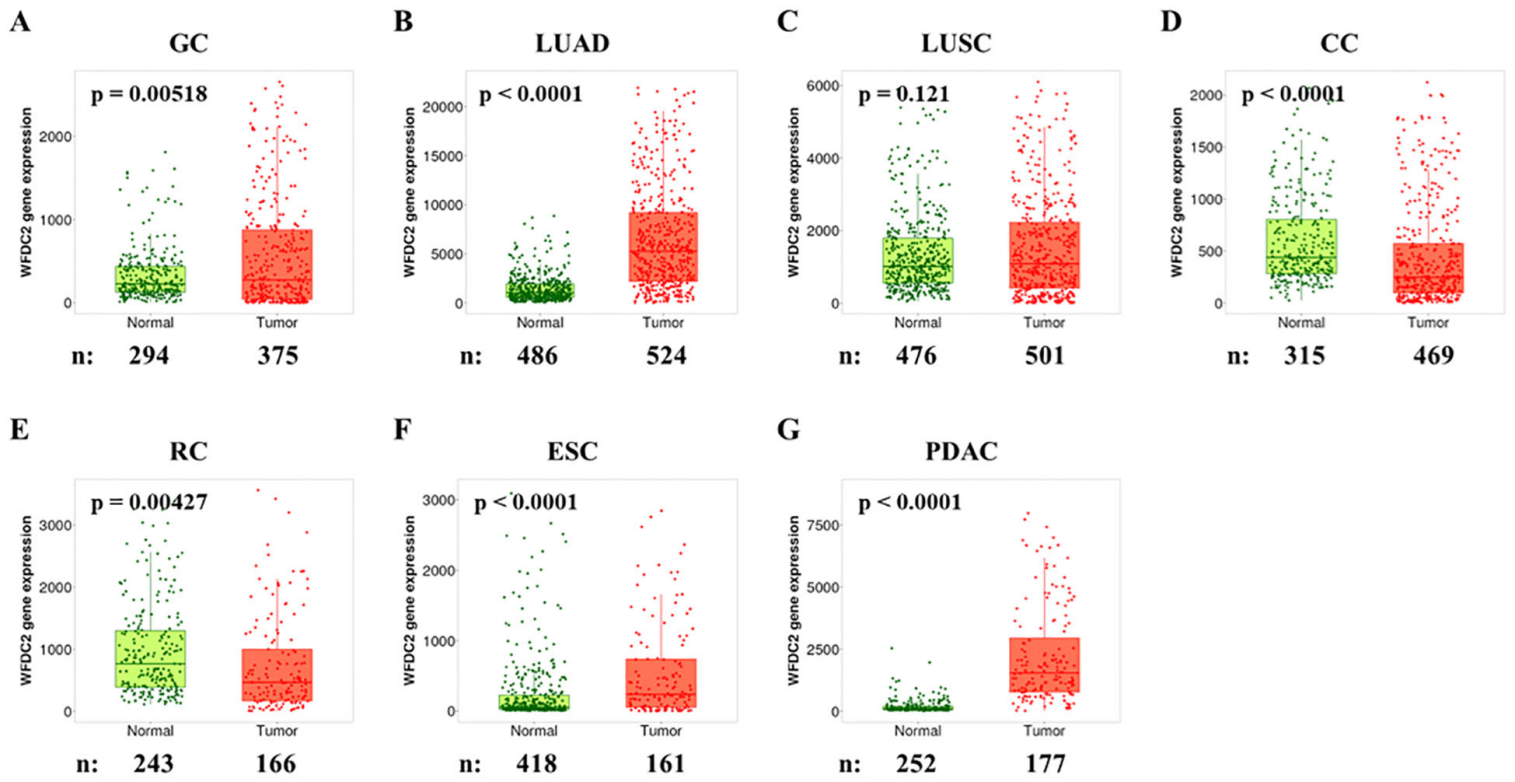
WFDC2 mRNA expression in normal and tumor tissues. Differential gene expression analysis of WFDC2 mRNA levels in normal and tumor tissues was conducted using data from the GEO, GTEx, TCGA, and TARGET databases through https://tnmplot.com/analysis/. **(A)** GC, gastric cancer; **(B)** LUAD, lung adenocarcinoma; **(C)** LUSC, lung squamous cell carcinoma; **(D)** CC, colon cancer; **(E)** RC, rectal cancer; **(F)** ESC, esophageal cancer; **(G)** PDAC, pancreatic ductal adenocarcinoma. Statistical significance was determined using the Mann-Whitney U test (*p < 0.05), and only samples with expression levels exceeding 10 in either tumor or normal tissues were included.

To compare WFDC2 mRNA levels between normal and tumor tissues across various cancer types, we analyzed publicly available data from the TNMplotter database (18) (**Figure 1A–G**). WFDC2 expression was significantly higher in gastric cancer, LUAD, esophageal carcinoma, and pancreatic ductal adenocarcinoma (PDAC) tumor tissues than in normal tissues (p < 0.01) (**Figure 1A, B, F, G**). Among these, LUAD tissues exhibited the most pronounced increase (p < 0.0001), whereas LUSC showed no significant difference (p = 0.21) (**Figure 1C**). WFDC2 expression was higher in LUAD than in LUSC, indicating a subtype-specific pattern in lung cancer. In contrast, WFDC2 expression was significantly lower in colon cancer and rectal cancer tumor tissues than in their corresponding normal tissues (p < 0.005) (**Figure 1D, E**). This suggests that WFDC2 expression patterns vary depending on the tissue type, with increased expression in certain epithelial tumors but decreased expression in colorectal malignancies. Overall, WFDC2 expression varied across different cancer types, exhibiting distinct patterns between adenocarcinomas and squamous cell carcinomas, as well as between gastrointestinal malignancies. Further investigation is required to determine the regulatory mechanisms governing these expression differences and their potential implications in tumor progression.

### 3.2 Association between WFDC2 mRNA expression and OS

**Figure 2:**
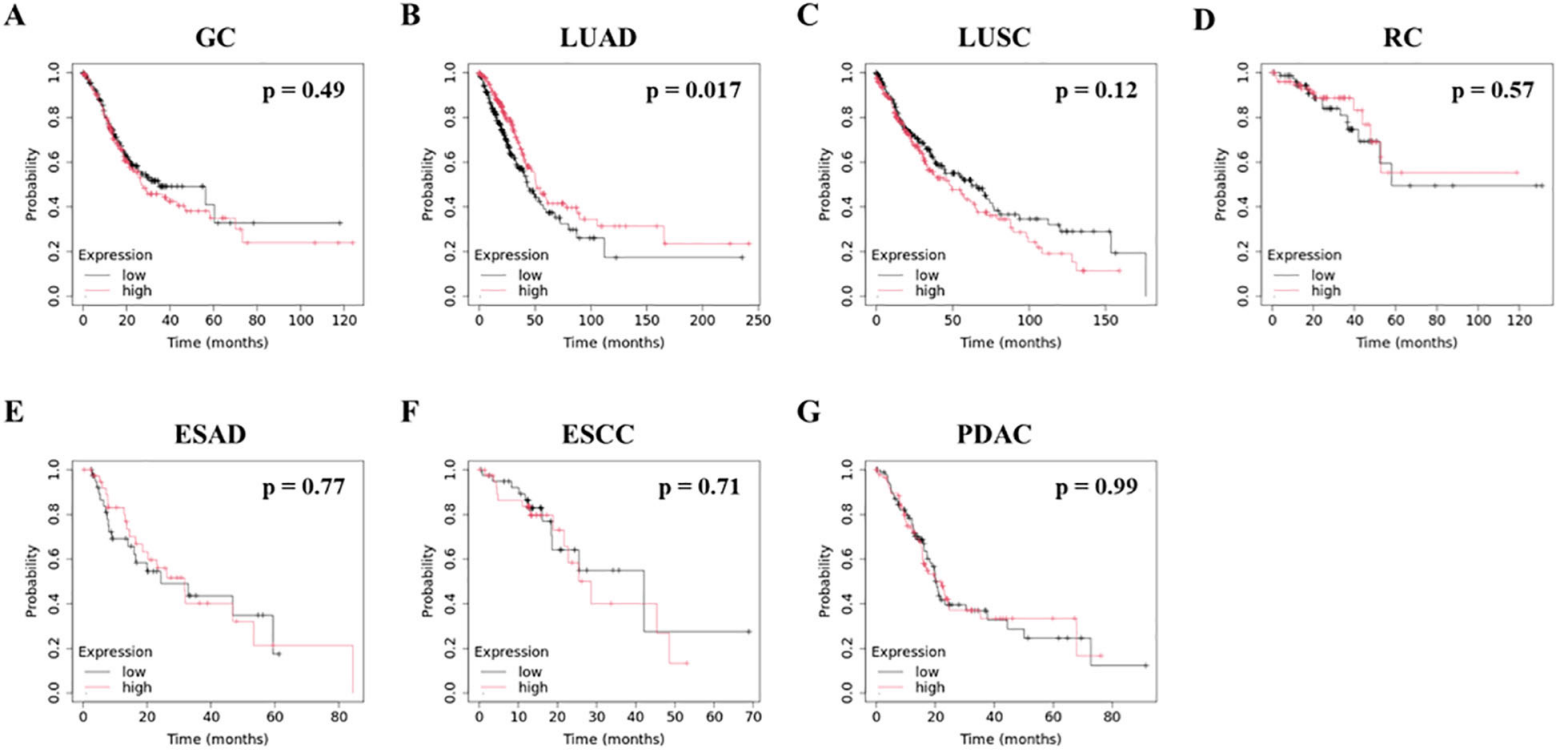
Association between WFDC2 mRNA expression and overall survival. Kaplan-Meier survival curves for overall survival were generated using data from TCGA, GEO, and EGA Series through http://KMplot.com. **(A)** GC, gastric cancer: high (n = 186), low (n = 185); **(B)** LUAD, lung adenocarcinoma: high (n = 252), low (n = 252); **(C)** LUSC, lung squamous cell carcinoma: high (n = 248), low (n = 247); **(D)** RC, rectal cancer: high (n = 82), low (n = 83); **(E)** ESAD, esophageal adenocarcinoma: high (n = 40), low (n = 40); **(F)** ESCC, esophageal squamous cell carcinoma: high (n = 40), low (n = 41); **(G)** PDAC, pancreatic ductal adenocarcinoma: high (n = 88), low (n = 89). Statistical significance was determined using the log-rank test, with p < 0.05 considered statistically significant.

Following this, we used the Kaplan-Meier Plotter to analyze the relationship between WFDC2 mRNA expression levels in tumor tissues and OS, utilizing data from three comprehensive databases: The Cancer Genome Atlas (TCGA), Gene Expression Omnibus (GEO), and European Genome-phenome Archive (EGA) (19). We examined multiple cancer types, including gastric cancer, LUAD, LUSC, rectal adenocarcinoma, esophageal adenocarcinoma (ESAD), esophageal squamous cell carcinoma (ESCC), and PDAC (**Figure 2A–G**). Elevated WFDC2 mRNA expression in LUAD tumor tissues was significantly associated with prolonged OS (p = 0.017) (**Figure 2B**), whereas no significant association was observed in LUSC (p = 0.12) (**Figure 2C**). This suggests a subtype-specific prognostic role of WFDC2 in lung cancer. In contrast, WFDC2 mRNA expression levels were not significantly correlated with OS in gastric cancer, rectal adenocarcinoma, ESAD, ESCC, or PDAC (p > 0.05 for all comparisons) (**Figure 2A, D–G**). These results indicate that WFDC2 expression in tumor tissues does not consistently predict OS across different cancer types, with LUAD being the only exception in our analysis. Collectively, these findings suggest that the prognostic significance of WFDC2 mRNA expression is dependent on the cancer type. The strong correlation observed in LUAD highlights the potential of WFDC2 as a biomarker for this subtype, whereas the lack of significant correlations in other cancers suggests that its prognostic value may be limited to specific tumor types.

### 3.3 Plasma WFDC2 levels and diagnostic performance in healthy donors and patients with cancer

**Figure 3:**
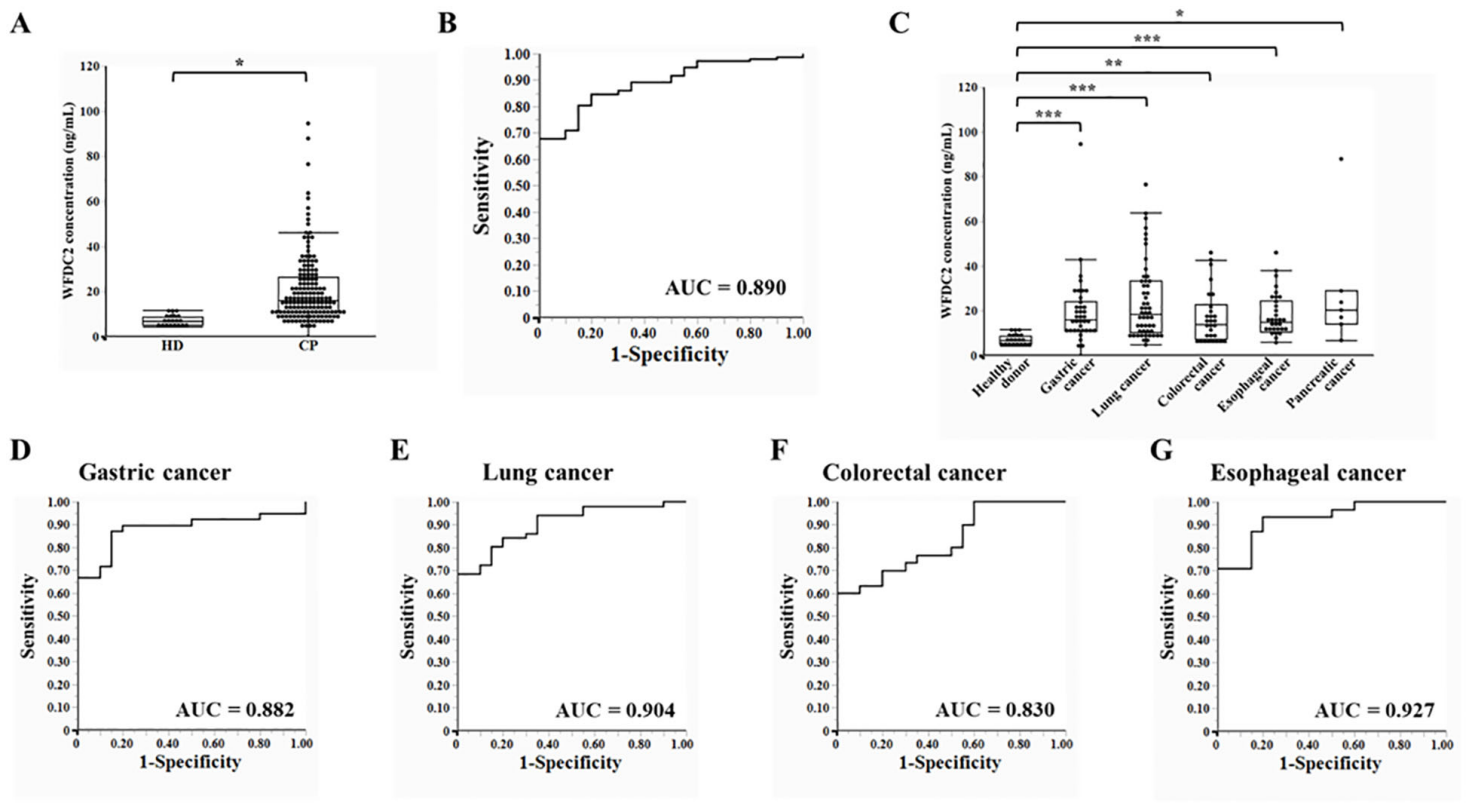
Plasma WFDC2 levels and diagnostic performance in healthy donors and patients with cancer. **(A)** Plasma WFDC2 levels measured using ELISA in healthy donors (n = 20) and patients with cancer (n = 158). Statistical analysis was performed using the Wilcoxon test. *indicates p < 0.0001. **(B)** ROC curve analysis of WFDC2 levels to distinguish patients with cancer from healthy donors. **(C)** Plasma WFDC2 levels by cancer type, including gastric cancer (n = 39), lung cancer (n = 51), colorectal cancer (n = 30), esophageal cancer (n = 31), and pancreatic cancer (n = 7), and in healthy donors (n = 20). Statistical significance was assessed using the Steel test. *indicates p < 0.005, ** indicates p < 0.0005, and *** indicates p < 0.0001. ROC curves for WFDC2 levels in gastric cancer **(D)**, lung cancer **(E)**, colorectal cancer **(F)**, and esophageal cancer **(G)** compared with those in healthy donors. ROC, receiver operating characteristic; AUC, area under the curve; HD, healthy donor; CP, patient with cancer. In **(A)** and **(C)**, a combination of dot plots and box plots is presented, with a markedly large outlier (colorectal cancer, 600.87 ng/mL) omitted to improve the clarity of the graph.

To evaluate the potential of WFDC2 as a plasma-based biomarker, ELISA was performed to measure plasma WFDC2 levels in both healthy donors and patients with cancer. Plasma WFDC2 concentrations were significantly elevated in patients with cancer compared with healthy donors (p < 0.0001, Wilcoxon test), suggesting its potential utility as a diagnostic biomarker (**Figure 3A**). To explore its diagnostic performance, ROC curve analysis was conducted. The area under the curve (AUC) for distinguishing patients with cancer from healthy donors was 0.890 (95% CI: 0.844–0.936) (**Figure 3B**). This high AUC value suggests the potential of WFDC2 as a reliable plasma biomarker for cancer detection.

Plasma WFDC2 levels were analyzed separately for each cancer type. WFDC2 concentrations were significantly higher in patients with gastric, lung, colorectal, esophageal, and pancreatic cancers compared to healthy donors (p < 0.01 for all comparisons, Steel test) (**Figure 3C**). ROC curve analysis revealed clear distinctions between healthy donors and cancer patients. In gastric cancer, the AUC was 0.882 (95% CI: 0.800–0.964), while in lung cancer, it was slightly higher at 0.904 (95% CI: 0.835–0.973). Colorectal cancer exhibited an AUC of 0.830 (95% CI: 0.726–0.934), whereas esophageal cancer showed the highest diagnostic performance, with an AUC of 0.927 (95% CI: 0.856–0.998) (**Figure 3D–G**). Collectively, the findings suggest that WFDC2 serves as a valuable marker for detecting multiple cancers. The diagnostic performance observed across various cancer types indicates its potential clinical utility. However, further validation in larger independent cohorts is necessary to confirm its applicability across different cancer subtypes and to establish its utility in clinical practice.

### 3.4 Association between plasma WFDC2 levels and OS

**Figure 4:**
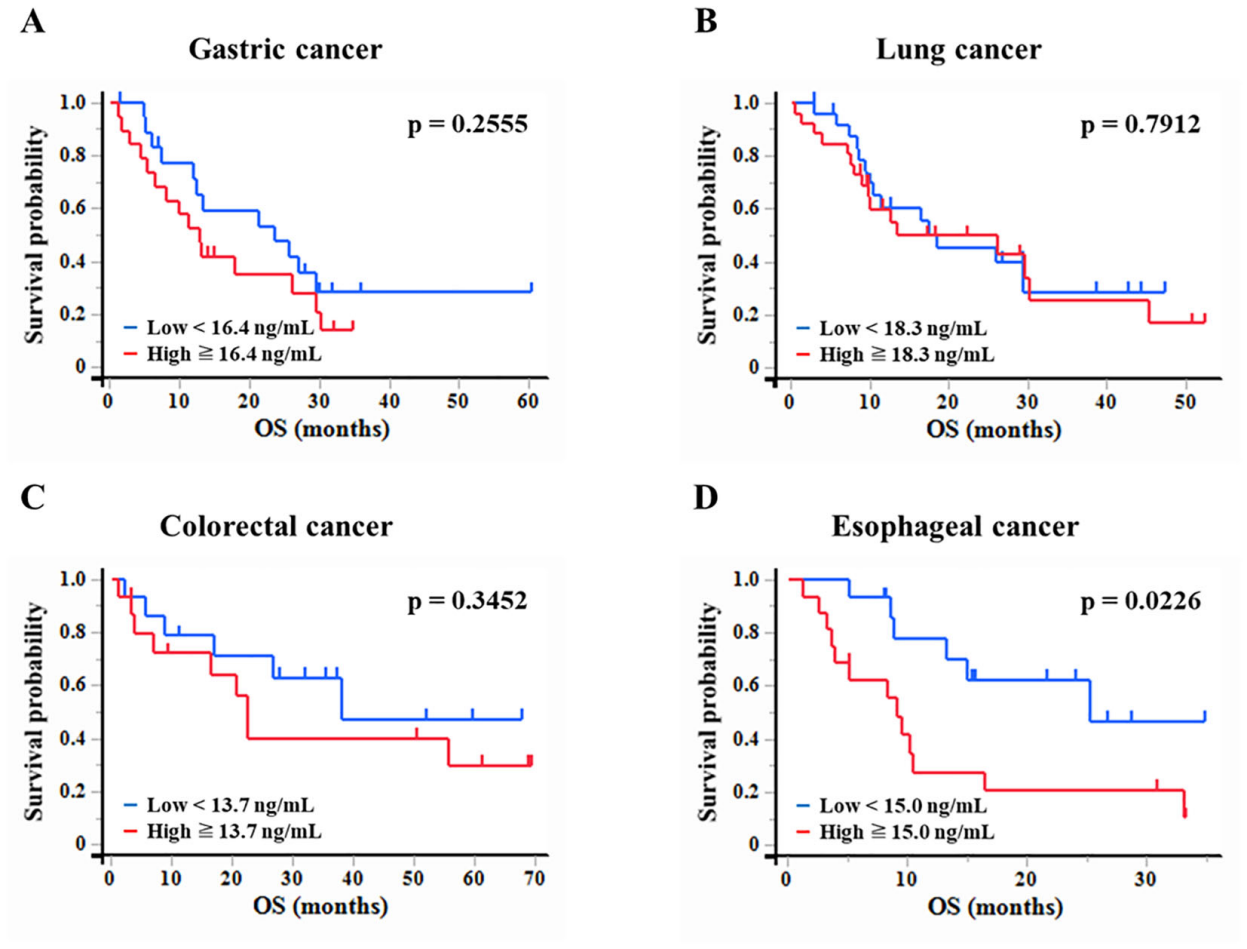
Association between plasma WFDC2 levels and overall survival. Kaplan-Meier survival analysis of overall survival in patients with cancer stratified by plasma WFDC2 expression levels. **(A)** Gastric cancer: low (n = 19), high (n = 20); **(B)** lung cancer: low (n = 25), high (n = 26); **(C)** colorectal cancer: low (n = 15), high (n = 15); and **(D)** esophageal cancer: low (n = 15), high (n = 16). Patients were divided into high and low WFDC2 expression groups based on the median plasma WFDC2 level. The red line represents the high WFDC2 expression group, whereas the blue line represents the low WFDC2 expression group. The log-rank test was used to assess differences in survival between the groups, with p < 0.05 considered statistically significant.

Kaplan-Meier survival analysis was performed to assess the association between plasma WFDC2 expression levels and OS in patients with gastric, lung, colorectal, and esophageal cancer (**Figure 4A–D**). Patients were classified into high and low WFDC2 expression groups based on the median plasma WFDC2 level, and survival differences were evaluated using the log-rank test. Although no significant differences in OS were observed between the high and low expression groups for gastric, lung, or colorectal cancer (**Figure 4A–C**), a significant difference was found in esophageal cancer (p = 0.0226), indicating a clear disparity in survival between the groups (**Figure 4D**). These results demonstrate that WFDC2 expression levels are associated with survival outcomes in esophageal cancer.

We examined the effect of clinical factors, including age, sex, body mass index, and performance status (PS), on OS (**Table 1**). In gastric cancer, univariate analysis revealed that WFDC2 concentration (HR = 1.04, 95% CI: 1.01–1.07, p = 0.005) and PS (HR = 2.46, 95% CI: 1.02–5.90, p = 0.044) were significantly associated with OS. Multivariate analysis identified WFDC2 concentration (HR = 1.04, 95% CI: 1.00–1.07, p = 0.019) as the sole independent prognostic factor (**Table 2**). In lung cancer, neither univariate nor multivariate analysis demonstrated a significant association between WFDC2 concentration and OS (**Table 3**). In colorectal cancer, univariate analysis showed that WFDC2 concentration (HR = 1.00, 95% CI: 1.00–1.01, p = 0.033) and PS (HR = 34.98, 95% CI: 3.08–397.00, p = 0.004) were significantly associated with OS; however, only PS (HR = 57.11, 95% CI: 2.58–1262.54, p = 0.010) remained significant in multivariate analysis (**Table 4**). In esophageal cancer, both WFDC2 concentration (HR = 1.07, 95% CI: 1.01–1.12, p = 0.009) and PS (HR = 7.30, 95% CI: 1.40–38.18, p = 0.018) were significantly associated with OS in univariate analysis and both remained independent prognostic factors in multivariate analysis (WFDC2: HR = 1.08, 95% CI: 1.02–1.13, p = 0.006; PS: HR = 7.70, 95% CI: 1.28–46.21, p = 0.026) (**Table 5**). Thus, WFDC2 concentration was identified as being independently associated with poor prognosis in both gastric and esophageal cancers based on multivariate analysis. Collectively, these findings suggest that WFDC2 plasma concentration is associated with OS in a cancer-type-specific manner and serves as an independent prognostic biomarker in specific cancer types.

**Table 1:** Demographic and clinical characteristics of the patients.

Characteristics				Healthy donor	Gastric cancer	Lung cancer	Colorectal cancer	Esophageal cancer
				n = 20	n = 39	n = 51	n = 30	n = 31
WFDC2 (ng/mL)				7.29± 2.37	20.01± 16.34	24.77± 19.57	28.52± 65.62	18.19± 9.96
Mean age (years)				43.30± 10.26	68.62± 10.45	71.49± 10.88	69.20± 10.41	68.29± 9.71
Sex	Male			11	29	37	16	28
	Female			9	10	14	14	3
BMI (kg/m^2^)					20.6± 4.20	22.54± 4.79	22.62± 3.34	20.55± 3.90
Performance status	0-1				31	47	28	29
	2-4				8	4	2	2
Stage	II				0	0	0	1
	III				1	4	4	0
	IV				38	47	26	30
Histological type	Adenocarcinoma	Stage	II		0	0	0	0
			III		1	2	4	0
			IV		38	31	26	0
	Squamous cell carcinoma	Stage	II		0	0	0	1
			III		0	1	0	0
			IV		0	11	0	30
	Others	Stage	II		0	0	0	0
			III		0	1	0	0
			IV		0	5	0	0

**Table 2:** Univariate and multivariate Cox regression analyses of prognostic factors for overall survival in gastric cancer.

Factor	Univariate		Multivariate	
	HR (95% CI)	p-value	HR (95% CI)	p-value
WFDC2 (ng/mL)	1.04 (1.01 – 1.07)	0.005	1.04 (1.00 – 1.07)	0.019
Age	0.98 (0.95 – 1.02)	0.313	1.00 (0.96 – 1.03)	0.787
Sex (Male vs. Female)	2.22 (0.84 – 5.89)	0.109	–	–
BMI (kg/m^2^)	1.05 (0.94 – 1.15)	0.387	–	–
Performance status (2–4 vs. 0-1)	2.46 (1.02 – 5.90)	0.044	1.70 (0.66 – 4.34)	0.270
Stage (III vs. IV)	NA	> 0.999	–	–

NA, not available.

**Table 3:** Univariate and multivariate Cox regression analyses of prognostic factors for overall survival in lung cancer.

Factor	Univariate		Multivariate	
	HR (95% CI)	p-value	HR (95% CI)	p-value
WFDC2 (ng/mL)	1.00 (0.98 – 1.02)	0.907	1.00 (0.98 – 1.02)	0.982
Age	1.00 (0.97 – 1.04)	0.861	1.00 (0.97 – 1.04)	0.870
Sex (Male vs. Female)	0.85 (0.39 – 1.83)	0.675	–	–
BMI (kg/m^2^)	1.00 (0.93 – 1.08)	0.911	–	–
Performance status (2–4 vs. 0-1)	1.17 (0.35 – 3.89)	0.793	1.16 (0.33 – 4.14)	0.815
Stage (III vs. IV)	0.68 (0.09 – 5.05)	0.708	–	–
Histological type (Adeno vs. others)	0.65 (0.32 – 1.33)	0.237	–	–

**Table 4:** Univariate and multivariate Cox regression analyses of prognostic factors for overall survival in colorectal cancer.

Factor	Univariate		Multivariate	
	HR (95% CI)	p-value	HR (95% CI)	p-value
WFDC2 (ng/mL)	1.00 (1.00 – 1.01)	0.033	1.00 (0.99 – 1.00)	0.569
Age	1.03 (0.97 – 1.09)	0.350	1.03 (0.97 – 1.10)	0.415
Sex (Male vs. Female)	0.85 (0.31 – 2.37)	0.762	–	–
BMI (kg/m^2^)	1.00 (0.87 – 1.19)	0.951	–	–
Performance status (2–4 vs. 0-1)	34.98 (3.08 – 397.00)	0.004	57.11 (2.58 – 1262.54)	0.010
Stage (III vs. IV)	NA	> 0.999	–	–

NA, not available.

**Table 5:** Univariate and multivariate Cox regression analyses of prognostic factors for overall survival in esophageal cancer.

Factor	Univariate		Multivariate	
	HR (95% CI)	p-value	HR (95% CI)	p-value
WFDC2 (ng/mL)	1.07 (1.01 – 1.12)	0.009	1.08 (1.02 – 1.13)	0.006
Age	1.00 (0.96 – 1.05)	0.965	0.98 (0.93 – 1.04)	0.492
Sex (Male vs. Female)	0.31 (0.06 – 1.49)	0.143	–	–
BMI (kg/m^2^)	1.00 (0.87 – 1.13)	0.972	–	–
Performance status (2–4 vs. 0-1)	7.30 (1.40 – 38.18)	0.018	7.70 (1.28 – 46.21)	0.026
Stage (II vs. IV)	NA	> 0.999	–	–

NA, not available.

## 4 Discussion

In this study, we investigated the prognostic potential of WFDC2 (HE4) across various cancer types. Our results revealed differences in WFDC2 expression between healthy individuals and patients with cancer, with its impact on OS varying depending on the cancer type. These findings suggest that WFDC2 serves as a valuable biomarker for cancer diagnosis and prognosis. However, as its effectiveness appears to be cancer-type dependent, further research is warranted to validate its reliability and broader applicability.

Database analysis showed significantly elevated WFDC2 mRNA expression in several cancer types, including gastric cancer, LUAD, esophageal cancer, and pancreatic cancer. A previous study reported a significant increase in WFDC2 expression in LUAD, while no significant difference was found in LUSC (12). This indicates that WFDC2 expression varies across different lung cancer subtypes. In contrast, WFDC2 expression was significantly lower in colorectal cancer, suggesting an inverse correlation between WFDC2 expression and cancer progression in some gastrointestinal cancer tissues. Immunostaining of ascitic fluid has revealed high WFDC2 positivity in ovarian cancer (91%), whereas gastric and colorectal cancers exhibit lower positivity rates (25% and 21%, respectively) (20). Hypoxia-induced upregulation of WFDC2 has been implicated in radiation therapy resistance in gastric cancer (21), with similar effects observed in colorectal cancer, where WFDC2 expression reduces radiation sensitivity (22). These findings suggest that distinct mechanisms underlie the role of WFDC2 in gastrointestinal cancers. However, studies analyzing WFDC2 levels in tissue or blood samples are limited; thus, it is difficult to draw definitive conclusions.

The prognostic impact of WFDC2 expression varies by cancer type, as indicated by the Kaplan-Meier analysis of database studies. In LUAD, high WFDC2 mRNA expression was significantly associated with prolonged survival, supporting its potential as a prognostic factor for this subtype. In contrast, no significant correlation was observed with LUSC, gastric, esophageal, or pancreatic cancers, indicating that WFDC2 plays different roles depending on cancer type and molecular characteristics. These discrepancies emphasize the need for cancer-type-specific therapeutic strategies. The elevated WFDC2 expression in LUAD may indicate a tumor microenvironment (TME) more responsive to therapeutic interventions, potentially explaining the improved survival observed in this subgroup.

A previous study comparing patients with lung cancer and healthy controls using serum ELISA reported an AUC of 0.988, indicating near-perfect diagnostic performance (23). In contrast, our plasma ELISA yielded an AUC of 0.904 for lung cancer, suggesting that plasma ELISA does not serve as a complete replacement for serum ELISA in diagnostic applications. Meta-analysis of WFDC2 in lung cancer demonstrated a pooled sensitivity of 0.73 and specificity of 0.86, with an area under the summary SROC curve of 0.86, supporting its potential as a diagnostic biomarker (24). In our study, the AUC for colorectal cancer was 0.830, which, although high, was lower than that for other cancer types. This suggests that WFDC2 is secreted into the bloodstream at lower levels from colorectal cancer tissue than from other cancer types. This contrasts with database analyses showing lower WFDC2 mRNA expression in colorectal cancer tissue than in normal tissue, despite elevated plasma WFDC2 concentrations in patients. This discrepancy highlights the need for further investigation into the mechanisms governing WFDC2 secretion within the TME. WFDC2 has been demonstrated to regulate the cytokine profile within the TME in ovarian cancer, promoting the polarization of tumor-associated macrophages toward the M2 phenotype while suppressing the infiltration and activation of NK cells and CD8^+^ T cells (25, 26). Furthermore, WFDC2 enhances PD-L1 expression in both tumor cells and macrophages, thereby facilitating immune evasion by impairing the function of immune effector cells (26). These findings suggest that WFDC2 plays a crucial role in suppressing antitumor immunity and promoting immune evasion, contributing to the establishment of an immunosuppressive TME.

To the best of our knowledge, there have been no reports of ELISA-based blood sample analysis in gastric cancer, and only a limited number of studies have explored serum ELISA in colorectal and esophageal cancers. These studies indicate low diagnostic value, with no reports on prognostic significance (16, 17). This study is the first to explore the utility of blood samples as biomarkers in gastric cancer using ELISA and determine the prognostic value of WFDC2 in gastric and esophageal cancers. We have demonstrated that WFDC2 serves as a prognostic factor in esophageal cancer, which is a key finding. In this study, we utilized plasma, which retains coagulation factors and better reflects circulating proteins, thus providing deeper insights into the role of WFDC2. Plasma processing is also faster as it does not require clotting, improving efficiency for high-throughput analysis. Considering the potential involvement of WFDC2 in immune modulation, plasma may offer a more physiologically relevant perspective. While serum remains a reliable and well-validated sample type for biomarkers such as WFDC2 (23, 24), plasma offers broader proteomic coverage and contributes to a more comprehensive understanding of the role of WFDC2 in cancer progression and its potential as a biomarker. In this study, although we initially planned to directly compare plasma WFDC2 levels and WFDC2 expression in tumor tissues assessed by immunohistochemistry (IHC), we were unable to perform this analysis due to an insufficient number of available tumor tissue samples. However, previous research from our laboratory, particularly in pancreatic cancer, has reported that high WFDC2 expression evaluated by IHC is associated with poor prognosis (15). These findings suggest the potential utility of WFDC2 as a prognostic marker. We believe that future studies examining correlations between plasma and tissue WFDC2 levels in cases where both blood and tissue samples are available would be highly valuable.

This study has several limitations. As part of our analysis, we also examined WFDC2 concentrations in relation to cancer stage across each cancer type. Our results showed no statistically significant differences in WFDC2 concentrations between stages I, II, and III (Wilcoxon p > 0.05; data not shown). This lack of significance is likely attributable to the limited number of patients in stages II and III, which reduced the statistical power to detect potential differences. Nevertheless, the consistent levels of WFDC2 across early to intermediate stages may indicate that its expression is established early in tumorigenesis. Further studies with larger, stage-stratified cohorts are needed to clarify whether WFDC2 levels correlate with cancer progression or reflect tumor burden in a stage-dependent manner. Additionally, combining WFDC2 with other biomarkers could enhance diagnostic and prognostic accuracy. Established markers such as CA125, which is widely used in ovarian cancer (8, 27), CEA, primarily utilized for colorectal cancer as well as lung adenocarcinoma, and CA19-9, mainly applied in pancreatic and other gastrointestinal cancers, may provide complementary information (17). Understanding cancer-type-specific molecular characteristics and differences in the TME is also crucial for comprehending the role of WFDC2 in cancer progression. Moreover, exploring the correlation between plasma WFDC2 levels and treatment response could provide valuable insights for developing targeted therapeutic strategies. Future studies should focus not only on protein expression but also on post-translational modifications, such as glycosylation, which regulate cancer development and progression, serve as crucial biomarkers, and provide targets for therapeutic interventions (28, 29). Understanding these mechanisms will be essential for enhancing the accuracy and clinical applicability of WFDC2 as a biomarker.

## 5 Conclusion

Plasma WFDC2 levels were significantly elevated in patients with cancer, indicating potential diagnostic utility across various cancers, including gastric, lung, colorectal, and esophageal cancers. Furthermore, elevated WFDC2 levels were associated with poor prognosis in gastric and esophageal cancers, highlighting its value as a prognostic biomarker. These findings highlight the clinical relevance of plasma WFDC2 in cancer diagnosis and prognosis.

## Data Availability

The datasets presented in this study can be found in online repositories. The names of the repository/repositories and accession number(s) can be found in the article/Supplementary Material.

## References

[1] Sung H Ferlay J Siegel RL Laversanne M Soerjomataram I Jemal A. Global cancer statistics 2020: GLOBOCAN estimates of incidence and mortality worldwide for 36 cancers in 185 countries. CA Cancer J Clin. (2021) 71:209–49. doi: 10.3322/caac.21660, PMID: 33538338

[2] Watterson A Coelho MA. Cancer immune evasion through KRAS and PD-L1 and potential therapeutic interventions. Cell Commun Signal. (2023) 21:45. doi: 10.1186/s12964-023-01063-x, PMID: 36864508 PMC9979509

[3] Malik MMUD Alqahtani MM Hadadi I Kanbayti I Alawaji Z Aloufi BA. Molecular imaging biomarkers for early cancer detection: A systematic review of emerging technologies and clinical applications. Diagn (Basel). (2024) 14:2459. doi: 10.3390/diagnostics14212459, PMID: 39518426 PMC11545511

[4] Kirchhoff C Habben I Ivell R Krull N. A major human epididymis-specific cDNA encodes a protein with sequence homology to extracellular proteinase inhibitors. Biol Reprod. (1991) 45:350–7. doi: 10.1095/biolreprod45.2.350, PMID: 1686187

[5] Kirchhoff C. Molecular characterization of epididymal proteins. Rev Reprod. (1998) 3:86–95. doi: 10.1530/ror.0.0030086, PMID: 9685187

[6] Hellstrom I Raycraft J Hayden-Ledbetter M Ledbetter JA Schummer M McIntosh M. The HE4 (WFDC2) protein is a biomarker for ovarian carcinoma. Cancer Res. (2003) 63:3695–700. 12839961

[7] Drapkin R Von Horsten HH Lin Y Mok SC Crum CP Welch WR. Human epididymis protein 4 (HE4) is a secreted glycoprotein that is overexpressed by serous and endometrioid ovarian carcinomas. Cancer Res. (2005) 65:2162–9. doi: 10.1158/0008-5472.CAN-04-3924, PMID: 15781627

[8] Moore RG McMeekin DS Brown AK DiSilvestro P Miller MC Allard WJ. A novel multiple marker bioassay utilizing HE4 and CA125 for the prediction of ovarian cancer in patients with a pelvic mass. Gynecol Oncol. (2009) 112:40–6. doi: 10.1016/j.ygyno.2008.08.031, PMID: 18851871 PMC3594094

[9] Lamy PJ Plassot C Pujol JL. Serum HE4: an independent prognostic factor in non-small cell lung cancer. PloS One. (2015) 10:e0128836. doi: 10.1371/journal.pone.0128836, PMID: 26030627 PMC4452338

[10] Li J Li J Hao H Lu F Wang J Ma M. WFDC2, and CXCL14 as candidate biomarkers for early diagnosis of lung adenocarcinoma. BMC Cancer. (2023) 23:110. doi: 10.1186/s12885-023-10523-z, PMID: 36721112 PMC9887767

[11] Min B Wang Y. WFDC2 is a potential prognostic and immunotherapy biomarker in lung adenocarcinoma. J Int Med Res. (2024) 52:3000605241258893. doi: 10.1177/03000605241258893.11, PMID: 39068532 PMC11287736

[12] Zhang T Chu L Tan W Ye C Dong H. Human epididymis protein 4, a novel potential biomarker for diagnostic and prognosis monitoring of lung cancer. Clin Respir J. (2024) 18:e13774. doi: 10.1111/crj.13774, PMID: 38742362 PMC11091784

[13] O’Neal RL Nam KT LaFleur BJ Barlow B Nozaki K Lee HJ. Human epididymis protein 4 (HE4) is upregulated in gastric and pancreatic adenocarcinomas. Hum Pathol. (2013) 44:734–42. doi: 10.1016/j.humpath.2012.07.017, PMID: 23084584 PMC3556378

[14] Guo YD Wang JH Lu H Li XN Song WW Zhang XD. The human epididymis protein 4 acts as a prognostic factor and promotes progression of gastric cancer. Tumour Biol. (2015) 36:2457–64. doi: 10.1007/s13277-014-2858-0, PMID: 25432133 PMC4428537

[15] Ohkuma R Yada E Ishikawa S Komura D Kubota Y Hamada K. High levels of human epididymis protein 4 mRNA and protein expression are associated with chemoresistance and a poor prognosis in pancreatic cancer. Int J Oncol. (2021) 58:57–69. doi: 10.3892/ijo.2020.5147, PMID: 33367933 PMC7721086

[16] Kemal Y Demirag G Bedir A Tomak L Derebey M Erdem D. Serum human epididymis protein 4 levels in colorectal cancer patients. Mol Clin Oncol. (2017) 7:481–5. doi: 10.3892/mco.2017.1332, PMID: 28894584 PMC5582450

[17] Liu SY Bilal MA Zhu JH Li SM. Diagnostic value of serum human epididymis protein 4 in esophageal squamous cell carcinoma. World J Gastrointest Oncol. (2020) 12:1167–76. doi: 10.4251/wjgo.v12.i10.1167, PMID: 33133384 PMC7579729

[18] Bartha Á Győrffy B. TNMplot.com: A web tool for the comparison of gene expression in normal, tumor and metastatic tissues. Int J Mol Sci. (2021) 22:2622. doi: 10.3390/ijms22052622, PMID: 33807717 PMC7961455

[19] Balázs G. Integrated analysis of public datasets for the discovery and validation of survival-associated genes in solid tumors. Innovation (Camb). (2024) 5:100625. doi: 10.1016/j.xinn.2024.100625, PMID: 38706955 PMC11066458

[20] Stiekema A Van de Vijver KK Boot H Broeks A Korse CM van Driel WJ. Human epididymis protein 4 immunostaining of Malignant ascites differentiates cancer of Müllerian origin from gastrointestinal cancer. Cancer Cytopathol. (2017) 125:197–204. doi: 10.1002/cncy.21811, PMID: 28199067

[21] Peng C Liu G Huang K Zheng Q Li Y Yu C. Hypoxia-induced upregulation of HE4 is responsible for resistance to radiation therapy of gastric cancer. Mol Ther Oncolytics. (2018) 6:49–55. doi: 10.1016/j.omto.2018.11.004, PMID: 30705965 PMC6348758

[22] Shi LP Guo HL Su YB Zheng ZH Liu JR Lai SH. MicroRNA-149 sensitizes colorectal cancer to radiotherapy by downregulating human epididymis protein 4. Am J Cancer Res. (2018) 8:30–8., PMID: 29416918 PMC5794719

[23] Iwahori K Suzuki H Kishi Y Fujii Y Uehara R Okamoto N. Serum HE4 as a diagnostic and prognostic marker for lung cancer. Tumour Biol. (2012) 33:1141–9. doi: 10.1007/s13277-012-0356-9, PMID: 22373583

[24] He YP Li LX Tang JX Yi L Zhao Y Zhang HW. HE4 as a biomarker for diagnosis of lung cancer: A meta-analysis. Med (Baltimore). (2019) 98:e17198. doi: 10.1097/MD.0000000000017198, PMID: 31574828 PMC6775374

[25] James NE Cantillo E Oliver MT Rowswell-Turner RB Ribeiro JR Kim KK. HE4 suppresses the expression of osteopontin in mononuclear cells and compromises their cytotoxicity against ovarian cancer cells. Clin Exp Immunol. (2018) 193:327–40. doi: 10.1111/cei.13153, PMID: 29745428 PMC6149960

[26] Rowswell-Turner RB Singh RK Urh A Yano N Kim KK Khazan N. HE4 overexpression by ovarian cancer promotes a suppressive tumor immune microenvironment and enhanced tumor and macrophage PD-L1 expression. J Immunol. (2021) 206:2478–88. doi: 10.4049/jimmunol.2000281, PMID: 33903172

[27] Tian Y Li X Zhang H Wang Y Li H Qin Q. Serum NLR combined with CA125 and HE4 improves the diagnostic and prognostic efficiency in patients with ovarian cancer. Front Oncol. (2024) 14:1494051. doi: 10.3389/fonc.2024.1494051, PMID: 39882448 PMC11776095

[28] Pinho SS Reis CA. Glycosylation in cancer: mechanisms and clinical implications. Rev Nat Rev Cancer. (2015) 15:540–55. doi: 10.1038/nrc3982, PMID: 26289314

[29] Thomas D Rathinavel AK Radhakrishnan P. Altered glycosylation in cancer: A promising target for biomarkers and therapeutics. Biochim Biophys Acta Rev Cancer. (2021) 1875:188464. doi: 10.1016/j.bbcan.2020.188464, PMID: 33157161 PMC7855613

